# Neutrophils and Neutrophil Extracellular Traps Regulate Immune Responses in Health and Disease

**DOI:** 10.3390/cells9092130

**Published:** 2020-09-20

**Authors:** Shrikant R. Mulay, Hans-Joachim Anders

**Affiliations:** 1 Division of Pharmacology, CSIR-Central Drug Research Institute, Lucknow 226031, India; 2 Division of Nephrology, Department of Medicine IV, Hospital of the LMU Munich, 80336 Munich, Germany

## 1. Introduction

Neutrophils are first responders of antimicrobial host defense and sterile inflammation, and therefore, play important roles during health and disease. Almost 16 years after the first description of neutrophil extracellular traps (NETs) as an alternative mode of pathogen killing, it has become clear that NETs also largely contribute to sterile forms of inflammation [1]. Indeed, NETs contribute to all forms of thrombosis, microparticle-induced inflammation, autoimmune vasculitis, auto-inflammatory disorders, and secondary inflammation due to ischemic, toxic, or traumatic tissue injury [1]. Recently, NETs have also been found to be an essential component of the multi-organ complications of COVID-19 [2].

In this Special Issue in *Cells*, we selected a series of articles that highlight the role of neutrophils and NETs in various sterile and non-sterile, acute and chronic inflammatory conditions affecting both human and animal health. We hope that this Special Issue will instigate novel research questions in the minds of our readers and will be instrumental in the further development of the field.

## 2. Neutrophils and NETs in Infection

Neutrophils play crucial roles in innate and adaptive immune responses. They control the invading pathogens, e.g., bacteria, fungi, and viruses, via multiple mechanisms, e.g., phagocytosis and NET formation. Phagocytosis of pathogens kills by exposing them to intracellular bactericidal compounds, whereas NET formation results in trapping and killing pathogens outside the cell.

In this Special Issue, Manda-Handzlik and Demkow discuss the emerging role of NETs in central nervous system infections [3]. They suggested netting neutrophils as the main causative factor for disruption of the blood–brain barrier integrity, subsequently leading to neuroinflammation and cell death [3]. Likewise, Appelgren et al. found the presence of NETs to strongly correlate with the presence of pleocytosis and neutrophil-stimulating cytokines/chemokines in cerebrospinal fluid samples collected from pediatric and adult patients with Lyme neuroborreliosis, Lyme disease, tick-borne encephalitis, enteroviral meningitis, and other viral infections [4].

Infection by *Plasmodium* during malaria results in the formation of the insoluble crystalline pigment hemozoin by digestion of hemoglobin in red blood cells. Rupture of red blood cells releases hemozoin crystals into the circulation from where it is usually cleared by neutrophils. A wide range of crystalline particles have been demonstrated to induce NETs formation [5,6,7]. In this issue, Lautenschlager et al. demonstrate that engulfment of hemozoin crystals by neutrophils is regulated by crystal–platelet interactions as well as plasma proteins such as fibrinogen, where the latter inhibits crystal uptake and clearance from the extracellular space [8]. Surprisingly, unlike many other crystalline particles, the ingestion of hemozoin crystals by neutrophils does not induce NETs formation [8]. Next, Estúa-Acosta et al. challenge the idea that the eye is an immune-privileged organ and provide evidence of NETs and their implication in pathophysiology in infectious keratitis, the leading cause of monocular blindness as well as non-infectious eye diseases [9]. Meanwhile, Magán-Fernández et al. summarize the current knowledge about the role of NETs in the pathogenesis of periodontitis [10]. Infection of the periodontium starts with the accumulation of a complex bacterial biofilm that induces dysbiosis between the gingival microbiome and the immune response of the host, which involves impaired NET formation and/or elimination [10]. Furthermore, the formation of biofilms promotes the efficient growth of pathogenic bacteria and fungi by providing optimal local environmental conditions and increased protection against the immune system [11]. The functional properties of the biofilm are regulated by a cell-to-cell communication system, called quorum sensing, which involves numerous quorum sensing-related molecules [11]. In this issue, Zawrotniak et al. define one quorum sensing molecule of *Candida albicans*, i.e., farnesol, that is capable of inducing NET formation, a process sensing the infection to the host immune system early and to limit spreading of the fungal infection [12].

The novel severe acute respiratory syndrome coronavirus (SARS-CoV)-2 infection, named COVID-19, is characterized by neutrophilia and increased neutrophil-to-lymphocyte ratio [13]. In this issue, we propose that continuous infiltration of neutrophils and NETs formation to the site of infection and at sites of organ injury drive necroinflammation and contribute to organ failure such as acute respiratory distress syndrome. NETs also contribute to cytokine storm and sepsis development as well as to the formation of endothelial injury and microvascular thrombosis during COVID-19 [14].

## 3. Neutrophils and NETs in Sterile Inflammation

Besides infections, neutrophils and NETs also play a critical role in the development of sterile inflammation in several chronic inflammatory conditions. In this issue, Manda-Handzlik and Demkow discuss the contribution of NETs in different pathological conditions affecting the central nervous system, e.g., trauma, neurodegeneration, and autoimmune diseases [3]. Estúa-Acosta et al. discuss the contribution of NETs in eye physiology, e.g., eye rheum formation as well as pathophysiological conditions, e.g., dry eye disease, corneal injuries like alkali burn, uveitis, diabetic retinopathy, and age-related macular degeneration [9]. Interestingly, the same mechanism was found to be responsible for uveitis in large animals. Fingerhut et al. demonstrate the presence of NETs in the vitreous body fluids derived from the eyes of horses with recurrent uveitis [15].

Furthermore, Bonaventura et al. emphasize the pathogenic roles of NETs in the initiation and progression of cardiovascular diseases e.g., acute myocardial infarction, diabetes, and obesity involving activation of the NLRP3 inflammasome and thrombosis [16]. They also highlight the need for standardized nomenclature and standardized techniques for NET assessment and novel therapies targeting NETs [16]. Neutrophils and NETs also contribute to the development of liver diseases [17,18]. In this issue, Zhou et al. decipher the molecular mechanisms of neutrophil chemotaxis and NETosis in murine chronic liver injury. They demonstrate that the cannabinoid receptor 1 mediates neutrophil chemotaxis and NETosis via the Gα_i/o_/ROS/p38 MAPK signaling pathway in liver inflammation [19]. Above and beyond, cancer cells have been shown to induce NETs formation to support metastasis [20]. Decker et al., in this issue, evaluated the correlation between NETosis and disease progression during head and neck cancer [21]. They observe neutrophils from head and neck cancer patients and show increased NETosis in patients at an early stage compared to that of late-stage or healthy controls. Therefore, elevated NETosis can be used as a biomarker for the prognosis of the disease [21]. Consistent with this, Fousert et al. also propose NETs as promising biomarkers for autoimmune diseases since they contribute to the development of autoimmunity by breaking self-tolerance [22]. They further conclude that therapeutics targeted at neutrophils and NETs will be beneficial for the treatment of inflammatory autoimmune diseases [22].

## 4. Perspective

It is becoming evident that studying NETs reveals novel insights into the pathogenesis of many diseases. Whether or not NETs can also be a potential therapeutic target remains unclear at this point. Animal studies have demonstrated that inhibiting certain signaling pathways can prevent NETs formation, can enhance NETs clearance, or at least, cleave the externalized chromatin, which accounts for many of the pathogenic effects of NETs. However, whether such interventions will prove efficacious and safe in human disease settings remains to be demonstrated. The first clinical trials are testing the effects of nebulized Dornase, a recombinant form of DNAse I that cleaves extracellular NETs chromatin, in mechanically ventilated patients with severe COVID-19 (NCT04432987, NCT04359654, NCT04402970) or severe trauma (NCT03368092) as an attempt to reduce NETs-related respiratory failure. Positive data of such trials may encourage clinical trials with NETs-targeting interventions also in other clinical domains. We hope that the readers of this *Cells* issue enjoy the scientific content and feel inspired to continue research in this evolving domain for a better understanding of disease and hopefully also, for better treatment options for NETs-related disorders in the future.
